# Antibiotic Prescription for Treatment and Prevention of Odontogenic Infections: A Cross-Sectional Survey of Lithuanian Dentists

**DOI:** 10.3390/medicina60111745

**Published:** 2024-10-24

**Authors:** Neringa Skucaite, Lukas Stundžia, Rita Veberiene, Vilma Brukiene, Vita Maciulskiene

**Affiliations:** 1 Clinic of Dental and Oral Pathology, Faculty of Odontology, Medical Academy, Lithuanian University of Health Sciences, LT-50161 Kaunas, Lithuania; neringa.skucaite@lsmu.lt (N.S.); lukas.stundzia@lsmu.stud.lt (L.S.); rita.veberiene@lsmu.lt (R.V.); 2 Institute of Odontology, Faculty of Medicine, Vilnius University, LT-03101 Vilnius, Lithuania; vilma.brukiene@mf.vu.lt

**Keywords:** antibiotic prescription, dental treatment, survey

## Abstract

*Background and Objectives*: The inappropriate use of antibiotics can lead to antimicrobial resistance. Overprescribing in dental practice has been reported. This study aimed to describe patterns of antibiotic prescription for treating and preventing odontogenic infections based on reports from Lithuanian dentists. *Materials and Methods*: Questionnaires were sent to all 4751 Lithuanian dentists registered in the database of the Lithuanian Dental Chamber who had consented to participate in surveys. The questionnaire addressed antibiotic prescription preferences for the treatment and prevention of various dental pathologies. The statistical analysis included chi-square tests and a factor analysis to evaluate prescription frequences in different clinical scenarios considering the respondents’ specialty and age. *Results*: Of 647 responses, 497 were from general dentists, 35 from oral surgeons, 40 from endodontists, 20 from periodontists, and 35 from prosthodontists. Respondents were grouped by age: A (≤35 years, n = 207), B (36–50 years, n = 224), and C (≥51 years, n = 209). Amoxicillin was the first-choice antibiotic for 81.1% of respondents (group A more frequently than other groups, *p* = 0.001). A 7-day treatment duration was preferred by 60.8%, while 33.6% chose 5 days. For patients allergic to β-lactam antibiotics, 63% preferred clindamycin. Over 90% cited acute apical abscess with systematic involvement as an indication for antibiotic prescription. A factor analysis of 18 clinical scenarios revealed prescription differences among dental specialists, oral surgeons, and periodontists prescribing antibiotics more frequently than general dentists and endodontists. For prophylaxis, 87.5% recommended antibiotics for patients at risk of infectious endocarditis after a cardiologist’s consultation (group C less frequently than other groups, *p* = 0.021). *Conclusions*: Lithuanian dentists generally prefer narrow-spectrum antibiotics for the treatment of odontogenic infections. There are notable differences in prescription patterns among dental specialists, with younger dentists showing a trend towards more rational antibiotic use.

## 1. Introduction

The inappropriate use of antimicrobials, including antibiotics, in humans, animals, and plants is the main factor contributing to the development of drug-resistant pathogens, leading to ineffective treatment of infectious diseases [1,2]. High rates of resistance to currently available antibiotics have been documented in many regions [1]. Ineffective antimicrobial medicines make infections harder to treat, leading to prolonged illness, higher healthcare costs, and increased mortality [3]. In 2019, antimicrobial resistance (AMR) involving a diverse set of pathogens was estimated to be directly responsible for millions of deaths worldwide [4].

To address this issue, the World Health Assembly adopted a global action plan on AMR [5]. This plan aims to enhance public awareness and understanding of AMR, implement preventive measures against infections, optimize the use of antimicrobials in human and animal health, and promote sustainable economic practices and the development of modern technologies in medicine.

Currently, trends in antibiotic consumption are monitored in many countries. The collected data are reported annually by the Global Antimicrobial Resistance and Use Surveillance System (GLASS) and the European Surveillance of Antimicrobial Consumption Network [1,2]. These reports show that antibiotic consumption varies significantly worldwide, with notable differences in antibiotic choice and levels of antimicrobial resistance among countries.

In Lithuania, the AMR burden is the 38th lowest when measured by the age-standardized mortality rate per 100,000 population associated with AMR across 204 countries [3]. From 2013 to 2022, the overall consumption of antibacterials for systemic use at the community level remained unchanged [2]. However, the ratio of broad- to narrow-spectrum antibiotic use in the community sector has shifted significantly, with an increasing trend observed in nine European Union (EU) countries, including Lithuania [2].

Although most dental diseases are of infectious origin, systemic antibiotic therapy in dental practice is typically reserved for acute clinical situations involving the spread of inflammation in periodontal tissues or mandibular/maxillary bones. Furthermore, antibiotics are only indicated as an adjunct to definitive treatment in cases when the systemic spread of odontogenic infection (severe swelling of surrounding tissues or/and elevated body temperature) is evident [6]. Antimicrobials are not recommended for treatment of chronic odontogenic infections, periimplantitis, pericoronitis without systemic involvement, or to prevent pain associated with pulpitis or surgical site infection after uncomplicated tooth extraction in healthy patients [6,7,8].

A key question regarding antibiotic therapy is to determine the indications and types of antibiotics suitable for a particular clinical situation. The literature suggests that dental practitioners often over-prescribe antibiotics [7]. Survey data from various countries have shown a lack of knowledge about antimicrobial therapy and prescribing, attributed to both patient- and clinician-related factors [7,8]. Published data on antibiotic prescribing patterns among Lithuanian dentists are limited to the treatment of endodontic infections [9,10]. Variations in antibiotic prescription trends based on years of professional experience have been observed [9,10]. However, these studies focused exclusively on general dental practitioners. Moreover, no information on antimicrobial therapy for surgical procedures, such as implant placement and tooth extraction, is available. The use of antibiotic prophylaxis for dental procedures in patients with systemic diseases has not been analyzed either.

The purpose of this study was to evaluate antibiotic prescription among Lithuanian dentists, considering various types of dental pathologies and treatment procedures among different specialists.

## 2. Materials and Methods

### 2.1. Study Sample and Data Collection

A cross-sectional survey was conducted from April 2024 to June 2024 among the practicing dentists in Lithuania. Bioethics authorization was granted by the Lithuanian University of Health Sciences Bioethics committee on 23 April 2024 (Certificate No: 2024-BEC3-T-013). Questionnaires regarding antibiotic prescriptions for various dental and oral diseases were sent to all 4751 dentists registered at the database of the Lithuanian Dental Chamber who had consented to take part in professional surveys. Respondents were contacted by e-mail and invited to anonymously fill in an online questionnaire hosted on the website https://manoapklausa.lt/ (accessed on 3 May 2024). During the study period, two reminders were sent out with a two-week interval.

The required sample size was calculated using the Paniotto Formula [11]. Thus, it was determined that 647 responses would be sufficient to draw valid conclusions, providing a 5% margin of error with a 95% confidence interval.

### 2.2. Questionnaire

The questionnaire comprised five questions with multiple-choice answers. The questionnaire was validated among the employees of the Clinic of Dental and Oral Pathology, Lithuanian University of Health Sciences, and refined for clarity and scope before distribution.

The questions addressed the respondent’s age, professional specialization, and attitudes regarding antibiotic prescription in clinical practice. Specifically, the respondents were asked to report their preferred antibiotic, its dosage, treatment duration, and indications for prescribing antibiotics for various odontogenic pathologies and antibiotic prophylaxis prior to certain dental procedures.

Four questions were structured with a possibility to select multiple answers, while the fifth question assessed the indications for prescribing antibiotics in 18 specific clinical scenarios using a five-point Likert scale (always, often, sometimes, seldom, or never). The respondents were asked to choose the option that best represented their clinical attitude by selecting only one category for this question (Questionnaire S1).

### 2.3. Data Analysis

The data were stratified by the age of respondents: group A (≤35 years), group B (36–50 years), and group C (≥51 years). The analysis also considered the specialty (general dentist, endodontist, oral surgeon, periodontist, prosthodontist, and others) of the respondents.

The statistical analysis involved chi-square tests to assess differences between age groups and specialties. Due to the significantly higher number of responses from general dental practitioners compared to other specialists, an additional analysis was conducted with 69 randomly selected participants from the general practitioners’ group to ensure reliability of the differences obtained.

A factor analysis was conducted to evaluate the frequency of antibiotic prescriptions in various clinical situations, considering both the specialty and age of the respondents. The obtained responses were grouped based on the five-point Likert scale. The factor analysis used a correlational matrix, with a main component method and VARIMAX rotation applied. The Kaiser–Meyer–Olkin (KMO) coefficient, which was 0.757, was calculated to assess the suitability of the matrix for factor analysis.

To evaluate the results of the factor analysis by specialty and age, an independent-sample Kruskal–Wallis’s test was performed.

## 3. Results

A total of 647 responses were received out of 4751 questionnaires sent to Lithuanian dentists, resulting in a response rate of 13.6%. Among the respondents, 497 were general dental practitioners, 35 were oral surgeons, 40 were endodontists, 20 were periodontists, 35 were prosthodontists, and 20 were other specialists (including pediatric dentists and orthodontists). Distribution of the respondents did not vary by age and was as follows: group A, 207; group B, 224; and group C, 209.

Amoxicillin was indicated by the majority of all respondents (81.1%, n = 519) as the most preferable antibiotic for treatment of odontogenic infections in patients without allergies to β-lactam antibiotics. The second choice was amoxicillin with clavulanic acid (52.6%, n = 333), with more than half of the respondents reporting that they prescribed this antibiotic.

The distribution of the responses regarding antibiotic choice for odontogenic infections with respect to dental specialty is presented in Table 1.

No significant differences between different specialists concerning the reported preferences of amoxicillin and amoxicillin with clavulanic acid for treatment of odontogenic infections were observed. Although other types of antibiotics were reported as being prescribed less frequently, clindamycin and metronidazole were mentioned significantly more often (*p* < 0.05) by periodontists (30%, n = 6) as compared to all other specialists. Azithromycin was prescribed more often by oral surgeons and periodontists than by other dentists. Additionally, 11.4% (n = 4) of oral surgeons indicated cefuroxime as their antibiotic of choice, while there were nearly no reports about it in other groups of specialists (Table 1).

A comparison of the results by the respondents’ age revealed significant differences in the prescriptions of amoxicillin and doxycycline. Specifically, dentists older than 35 years prescribed amoxicillin significantly less often (*p* = 0.001) compared to their young colleagues. Conversely, practitioners older than 50 years reported prescribing doxycycline more frequently than other respondents (*p* = 0.001) (Table 2).

An analysis of the data regarding the usually selected dosage of the preferred antibiotics indicated that amoxicillin 1000 mg twice per day and amoxicillin/clavulanate 875 + 125 mg twice per day were chosen by 70.2% (n = 454) and 46.2% (n = 299) of all respondents, respectively. Although amoxicillin 500 mg three times per day was chosen by a small number of the respondents (n = 82), this dose was preferred significantly more often by endodontists (32.5%, n = 13) and periodontists (35%, n = 7) compared to other dental specialists (*p* = 0.001).

A seven-day course of antimicrobial treatment was preferred by 60.6% (n = 392) of all respondents, while 33.4% (n = 216) preferred a five-day course. Endodontists reported prescribing a seven-day course significantly less frequently, while oral surgeons preferred it more often than other dental specialists (*p* < 0.05). Additionally, endodontists and prosthodontists indicated a preference for a five-day course more often, whereas periodontists and oral surgeons prescribed this less frequently than other groups (*p* < 0.05). The option of prescribing antibiotics until symptoms disappear was preferred by 10% (n = 4) of endodontists and significantly less by other specialists (Table 3).

Dentists younger than 36 years preferred the seven-day course of antimicrobial treatment significantly more often and the five-day course significantly less often compared to their older colleagues (Table 4).

Clindamycin was the most preferred antibiotic for the treatment of odontogenic infections in patients with allergies to penicillin (β-lactam antibiotics). More than half (62.6%, n = 405) of all respondents reported prescribing clindamycin. Although other types of antibiotics were reported as being prescribed less frequently than clindamycin, erythromycin was chosen significantly more often by oral surgeons and prosthodontists compared to other dental specialists (*p* < 0.05) (Table 5). In addition, 7.6% (n = 57) of all respondents reported prescribing various other antibiotics for patients with β-lactam allergies. Among these, 3.5% (n = 2) indicated doxycycline, 8.9% (n = 5) indicated cefuroxime, and 5.3% (n = 3) indicated lincomycin. The remaining respondents either did not know which antibiotic to prescribe or chose to consult a general physician.

The data analysis revealed that dentists younger than 36 years prescribed clindamycin significantly more often than those in other age groups (*p* = 0.001) (Table 6). In contrast, azithromycin was significantly more preferred by dentists in groups B and C compared to those in group A (*p* < 0.05). Tetracycline was chosen by 4.1% (n = 26) of all respondents and significantly more often by dentists older than 51 years compared to other age groups (*p* = 0.001).

**Figure 1:**
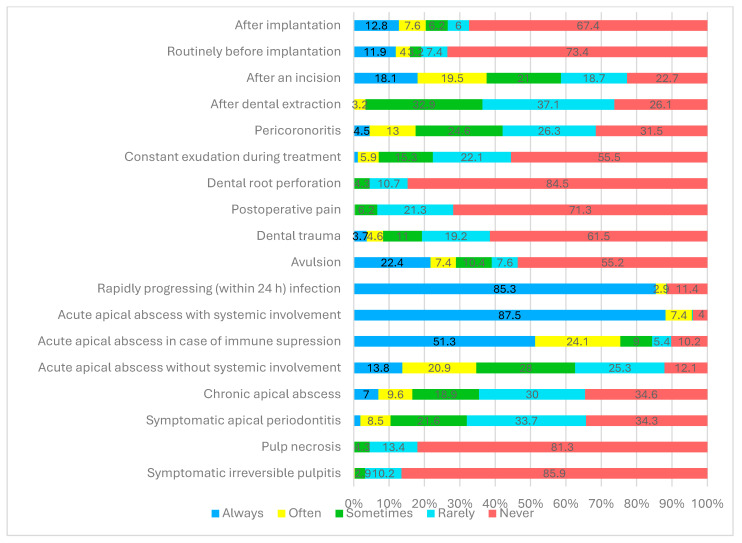
Prescription of antibiotics according to the type of dental pathology and particular dental procedures, as reported by Lithuanian dentists.

The distribution of the responses regarding antibiotic prescription for specific clinical scenarios in dental pathology and treatment procedures is presented in Figure 1.

Over 90% (n = 624) (87.5% always, 7.4% often) of all respondents reported prescribing antibiotics in cases of acute apical abscess with systemic involvement (elevated body temperature > 38 °C, lymphadenopathy), and 85.3% (n = 552) always prescribed antibiotics in cases of rapidly progressing (within 24 h) infection.

More than 70% (n = 448) of respondents (51.3% always, 24.1% often) prescribed antibiotics for the treatment of acute apical abscess in immunocompromised patients. About one-third (29.8%, n = 193) of all respondents considered prescribing antibiotics in cases of avulsion and 15.9% (n = 103) and 20.4% (n = 141) did so before and after dental implantation procedures.

Symptomatic irreversible pulpitis and pulp necrosis were reported as indications for antibiotic therapy by less than 5% (n = 12) of all respondents. The attitudes of the dental practitioners regarding antibiotic prescription in cases of tooth extraction, pericoronitis, incision procedures, symptomatic apical periodontitis, and chronic apical abscess varied widely, with less than 35% of all respondents never prescribing antibiotics in these clinical situations.

Based on the factor analysis, the responses were grouped into six factors based on the frequency of answers:

- Factor one: Symptomatic irreversible pulpitis, pulp necrosis, postoperative pain, perforation of the root, and constant exudation from root canals during endodontic treatment.
- Factor two: Chronic apical abscess, acute apical abscess without systemic involvement, and symptomatic apical periodontitis.
- Factor three: Routine prophylaxis before and after implantation.
- Factor four: Acute apical abscess in cases of immune suppression and acute apical abscess with systemic involvement and rapidly progressing (within 24 h) infection.
- Factor five: Pericoronitis post-tooth extraction and post-incision.
- Factor six: Avulsion and dental trauma.

**Figure 2:**
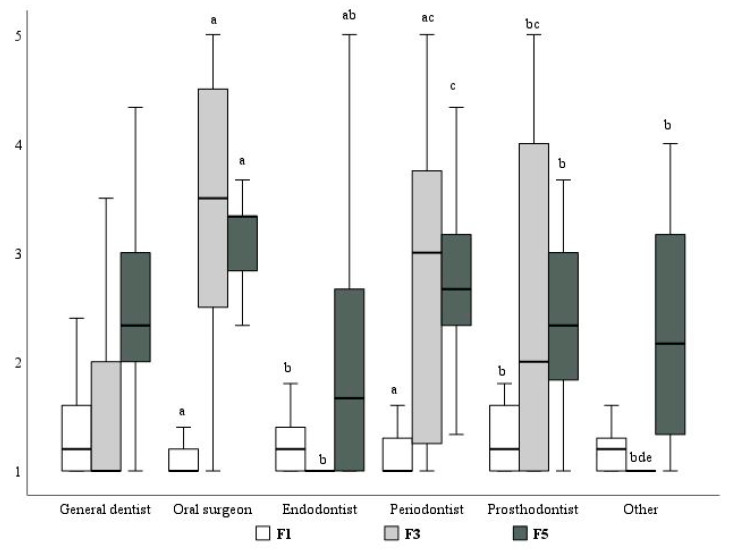
Factor analysis of antibiotic prescription in particular clinical situations by dental specialties: a—value significantly different from general dentists; b—value significantly different from oral surgeons; c—value significantly different from endodontists; d—value significantly different from periodontists; e—value significantly different from prosthodontists (*p* < 0.05).

The factor analysis of the eighteen clinical scenarios presented in the questionnaire (question 5) showed that oral surgeons reported prescribing antibiotics for clinical situations assigned to factor 1 less frequently than general dentists, endodontists, and prosthodontists, while periodontists did it less frequently than general dentists (Figure 2).

In clinical situations assigned to factor 3, oral surgeons and periodontists reported prescribing antibiotics significantly more often than general dentists and endodontists. Prosthodontists prescribed antibiotics more frequently than endodontists but less frequently than oral surgeons (Figure 2).

In clinical situations assigned to factor 5, endodontists prescribed antibiotics significantly less frequently than general dentists, periodontists, and oral surgeons. Conversely, oral surgeons prescribed antibiotics more often than other specialists, except for periodontists.

**Figure 3:**
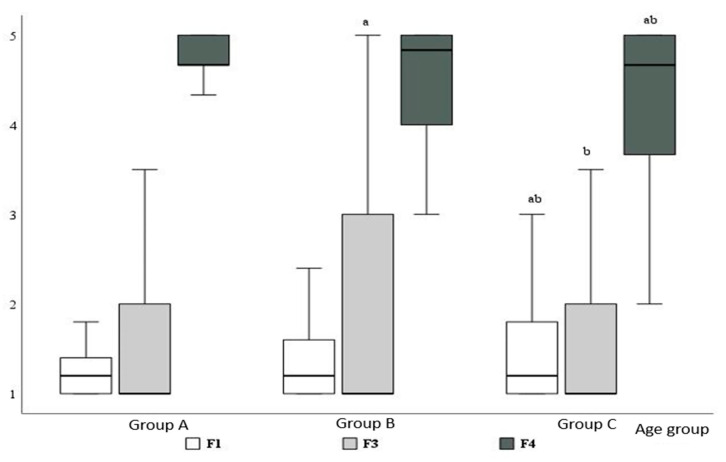
Factor analysis of antibiotic prescription in specific clinical situations by respondents’ age groups: a—significantly different from age group A; b—significantly different from age group B (*p* < 0.05).

No significant differences were observed in antibiotic prescriptions across dental specialties for clinical situations associated with factors 2, 4, and 6. When evaluating antibiotic prescription frequency in relation to respondents’ age, significant differences were observed for factors 1, 3, and 4 (Figure 3).

Thus, dentists older than 51 years prescribed antibiotics more frequently in clinical situations assigned to factor 1 and less frequently in situations assigned to factor 4 compared to their younger colleagues. Dentists aged 36–50 years reported prescribing antibiotics significantly more often than other age groups in cases assigned to factor 3. No significant differences were observed for other factors with respect to the respondents’ age.

Regarding antibiotic prophylaxis administered for dental procedures, most dentists prescribed antibiotics in cases of immune suppression due to systemic pathology when recommended by a treating physician and for patients at risk for infectious endocarditis (IE) after a consultation with a cardiologist (76% (n = 492) and 87.5% (n = 566), respectively). (Table 7).

Oral surgeons prescribed antibiotics for patients who have undergone arthroplasty within 3 months significantly more often than other specialists. Similarly, oral surgeons and periodontists prescribed antibiotics significantly more frequently than other dental practitioners for patients undergoing radiotherapy and intravenous bisphosphonate treatment (*p* < 0.05).

The older respondents (group C) prescribed antibiotics for prophylaxis during dental procedures significantly less frequently than their younger colleagues. This included prescriptions for patients with immune suppression due to systemic pathology when recommended by a physician, for patients at risk of IE, and for those after arthroplasty within the last three months (*p* < 0.05). Additionally, group B prescribed antibiotics for patients after arthroplasty within the last 3 months significantly less frequently than group A (*p* = 0.001) (Table 8).

## 4. Discussion

The World Health Organization recommends that antimicrobial treatments be evidence-based, using the narrowest spectrum of antibiotics, an appropriate dosage, minimal effective duration of therapy, and preferably a single antibiotic guided by microbiology [1].

The present survey revealed that the Lithuanian dentists most commonly prescribed amoxicillin or amoxicillin/clavulanate for treatment of odontogenic infections. This practice aligns with clinical recommendations [6,12,13] and reflects a global trend in antibiotic prescription by dentists [14,15,16,17,18,19,20]. Interestingly, the pattern of antibiotic prescribing among Lithuanian dentists has remained consistent over the past 13 years, as similar preferences were reported in previous surveys conducted in Lithuania [9,10]. Unlike some countries where broader-spectrum amoxicillin/clavulanate is preferred, Lithuanian dentists indicated amoxicillin as the first-choice antibiotic for treatment of dental infections [14,16,19,21]. The present study demonstrated that clindamycin, metronidazole, and azithromycin, although rarely prescribed in dental practice, were chosen more frequently by oral surgeons and periodontists than by other dental specialists. The choice of less common antibiotic types could possibly be dictated by specific infection types in periodontology and oral surgery.

Age-related differences in antibiotic preferences were observed. For example, doxycycline, a broad-spectrum antibiotic, was indicated by 5% of the dentists older than 50 years, while it was rarely reported among younger groups. Although preferences for amoxicillin/clavulanate choice did not differ between three age groups of the respondents, the younger dentists preferred the narrow-spectrum amoxicillin significantly more frequently. This trend mirrors the findings in Lithuania from four years ago but contrasts with a previous survey 13 years ago, where prescriptions of broader-spectrum antibiotics by younger dentists were reported [9,10]. Amoxicillin and amoxicillin/clavulanate are classified as “Access” group antibiotics according to the WHO AWaRe classification and are recommended to make up at least 65% of all antibiotic consumption at the country level [1,2]. Lithuania meets this criterion, with “Access” antibiotics comprising over 69% of total consumption since 2019 [2]. Considering that the prescription of antibiotics in dentistry constitutes a significant part of antibiotic consumption in medicine, the observed prescribing patterns among Lithuanian dentists likely contribute to the national antibiotic consumption.

Clindamycin was the most preferable antibiotic for patients allergic to penicillin-group antibiotics, consistent with recommendations [6,12,13] and international practices [14,15,16,17,18,19,20].

Most respondents preferred 1000 mg of amoxicillin or 875/125 mg of amoxicillin/clavulanate twice per day. Interestingly, there was a tendency to prescribe smaller doses of amoxicillin (500 mg) three times per day among endodontists and periodontologists, possibly following the recommendations of the European Society of Endodontology (ESE) [6].

The survey found that 61% of respondents preferred a 7-day course of antibiotics, while one-third preferred a 5-day course. This suggests that Lithuanian dentists may not fully adhere to recommendations for the shortest effective antimicrobial treatment duration [6,22]. Similar trends toward longer-course prescriptions have been observed in a few other countries as well [14,19]. The overuse of antibiotics is a major factor in resistance development, which correlates with exposure duration [22]. Furthermore, there is increasing evidence that the development of antibiotic resistance directly correlates with the duration of exposure [22]. Based on the present survey, endodontists and prosthodontists tend to prescribe antibiotics for a shorter period of time than other dentists. The trend toward a shorter course of antibiotic therapy was also observed among older respondents compared to their younger colleagues. It could be speculated that more severe infections encountered in oral surgery have influenced the choice to prescribe longer courses of antimicrobial treatment.

An important aspect in antimicrobial therapy is to avoid prescriptions of antibiotics in the absence of clinical indications. The survey indicates that Lithuanian dentists generally prescribe antibiotics appropriately for endodontic infections. Most dentists, regardless of their specialty, prescribe antibiotics only in cases of acute apical abscess with systemic involvement and rapidly progressing (within 24 h) infection. Such an attitude aligns with the ESE guidelines regarding the use of antibiotics in endodontics [6]. Nearly no reports about the need for antimicrobial treatment of symptomatic pulpitis and pulp necrosis were observed, in contrast to the results of a few surveys in other countries where such a tendency was reported [14,16,19]. Nevertheless, some knowledge gaps regarding indications for antibiotic therapy in the case of endodontic infection were observed. More than 60% of the respondents reported prescribing antibiotics in cases of chronic apical abscess and symptomatic apical periodontitis, although these diseases are not considered an indication for antibiotic therapy [6]. As the definition of chronical apical abscess does not exist in the Lithuanian classification of endodontic pathology, it could be erroneously interpreted as acute abscess and, therefore, lead to inappropriate treatment choice. Thus, careful revision of the local classifications of dental pathologies and their correlation with the international classification can be recommended.

Antibiotic prescribing practices varied among Lithuanian dentists for surgical interventions such as tooth extraction, incision, and implantation. Only 4% of the respondents prescribed antibiotics routinely after tooth extraction, while 70% did so seldomly or sometimes. It is important to note that the questionnaire did not allow the respondents to specify the clinical situations leading to tooth extraction; therefore, the obtained responses were rather general. The evidence regarding indications for antibiotics to prevent the risk of infection after tooth extraction is mixed, with some studies reporting reducing the infection risk [23] and others finding no additional benefits of antibiotics in healthy patients [24]. Obviously, extractions of teeth can be performed for various reasons and are related to the varying magnitude of the infection risk (e.g., extraction of impacted wisdom teeth, simple extraction due to caries or periodontal diseases in healthy people or in those with systemic pathology, number of teeth extracted during one visit, etc.). Moreover, the standard protocols of antibiotic prescription (before or after tooth extraction) could vary as well [25]. Thus, the data regarding antibiotic prescription for tooth extraction should always be interpreted in relation to the main diagnosis and clinical situation rather than to the intervention itself.

For pericoronitis, despite no option for systemic involvement in the questionnaire, many dentists still prescribed antibiotics, reflecting global trends [8,15,16]. Regarding dental implantations, 16% of Lithuanian dentists prescribed antibiotics before implantation and 20% after, consistent with international practices but with varying evidence for its efficacy [20,26]. Khouly et al. (2019) [26], based on a meta-analysis of 10 long-term clinical studies, concluded that antibiotic prophylaxis did not reduce the postoperative pain and infection risk and therefore was not necessary for the prevention of implant failure in healthy patients. In contrast, another meta-analysis [20] found sufficient evidence to suggest that a single-dose antibiotic prescribed preoperatively may reduce the occurrence of implant failures. The only clinical recommendations available in the world suggest prescribing 2 or 3 g of amoxicillin 1 h before the placement of dental implants without anatomical constraints, i.e., without the need to perform simultaneously regenerative procedures, in healthy patients, although non-prescription in certain cases could not be considered a wrong approach either [12]. Again, an analysis of the preventive antibiotic treatment should always be performed with caution, taking the individual patient characteristics as well as the need for additional interventions, such as regenerative surgery, into account. The respondents in the present survey did not have an option to evaluate clinical situations of implantation procedures, as the questions were focused exceptionally on the course (before or after implantation) of antibiotic therapy.

In dental trauma, the scientific evidence about the benefits of systemic antibiotics after replantation of the avulsed teeth is limited [27]. While over 50% of the Lithuanian dentists reported never prescribing antibiotics for tooth avulsion, about 20% did so routinely. In comparison, almost half (49%) of Italian dental practitioners prescribed antibiotics for replantation of the avulsed permanent teeth [28]. Such variations in antimicrobial treatment decisions can be associated with different recommendations published internationally. Thus, the International Association of Dental Traumatology recommends the use of systemic antibiotics after replantation of the avulsed teeth to prevent infection-related reactions and to reduce the risk of inflammatory root resorption [27], while the international guidelines for antimicrobial prescribing in dentistry suggest that antibiotics should not be considered routinely for avulsed teeth without signs of systemic infection due to the lack of scientific evidence [13].

According to the present survey, the knowledge of Lithuanian dentists regarding antibiotic prophylaxis administered before dental procedures is appropriate regarding patients with immune suppression due to systemic pathology or at risk of IE. The analysis of other indications for antibiotic prophylaxis showed that oral surgeons and periodontologists prescribed antibiotics in particular clinical situations (in patients undergoing head and neck radiotherapy or bisphosphonate therapy or in patients after arthroplasty) more often than any other dentists, possibly due to the higher frequency of such patients in oral surgery departments. Furthermore, the knowledge regarding antibiotic prophylaxis was related to the years after graduation of university, as older dentists reported prescribing antibiotic prophylaxis during dental procedures significantly less often than their younger colleagues. Similar patterns were observed in other published studies, showing that increasing age and time spent in practice could have a negative impact on the knowledge of respondents [29].

The factor analysis performed in this study demonstrated that oral surgeons and periodontists prescribed antibiotics more frequently than general dentists and endodontists before and after implantation procedures as well as in cases of pericoronitis, after tooth extraction, and after incision. Obviously, these results could be explained by the fact that implantation and other surgical procedures are part of routine practice in oral surgery and periodontology. On the contrary, oral surgeons reported prescribing antibiotics for some clinical situations related to endodontic treatment significantly less frequently than general dentists, endodontists, and prosthodontists, most likely because they did not perform endodontic treatment at all. Similarly, the data analysis with respect to the respondents’ age showed that the middle-age dentists (36–50 years) dominated among the respondents who prescribed antibiotics for implantation procedures, possibly due to the fact that implantation was performed most commonly by the dental professionals in this age group.

The small sample of dental specialists can be considered the main limitation of this survey. In order to perform comparisons, an additional analysis was performed using a selected group of general dentists, which allowed us to make assumptions regarding the prescription of antibiotics in different dental specialties. Another limitation of this survey is that some questions presented in the questionnaire were insufficiently detailed to obtain a clear answer, as discussed above in this manuscript. Therefore, the reported indications for antibiotic prescription in certain clinical situations should be interpreted with caution, and only trends could be discovered.

## 5. Conclusions

In conclusion, Lithuanian dentists generally adhere to the international recommendations for the use of antibiotics. The observed differences in the prescription patterns between different dental specialists can be attributed to the specific characteristics of dental pathology and guidelines provided by professional organizations. There is a tendency toward more rational use of antibiotic therapy among younger dentists, indicating the need for sharing the current knowledge about antimicrobial treatment in all dental communities.

## Figures and Tables

**Table 1:** Antibiotic choice for treatment of odontogenic infection as reported by Lithuanian dentists by their specialties.

Antibiotic	General Dentist % (n)	Oral Surgeon % (n)	Endodontist % (n)	Periodontist % (n)	Prosthodontist % (n)	Other % (n)	Of All Respondents % (n)
Amoxicillin	82.3 (409)	74.3 (26)	80 (32)	75 (15)	80 (28)	75 (15)	81.1 (525)
Amoxicillin with clavulanic acid	52.3 (260)	60 (21)	47.5 (19)	55 (11)	60 (21)	40 (8)	52.6 (340)
Clindamycin	2.4 (12)	5.7 (2) ^a^	5 (2) ^a^	30 (6) ^b^	8.6 (3) ^c^	0 (0) ^a^	3.9 (25)
Doxycycline	1.6 (8)	5.7 (2) ^a^	0 (0)	0 (0) ^a^	2.9 (1) ^a^	10 (2) ^b^	2 (13)
Azithromycin	2.4 (12)	14.3 (5) ^a^	0 (0)	10 (2) ^a^	2.9 (1) ^b^	0 (0) ^b^	3.1 (20)
Metronidazole	2.4 (12)	5.7 (2)	0 (0)	30 (6) ^a^	2.9 (1)	2.9 (1)	3.2 (21)
Cefuroxime	0.4 (2)	11.4 (4) ^a^	0 (0)	0 (0) ^b^	2.9 (1) ^b^	10 (2) ^a^	1.4 (9)

Within the same row, values with different superscript letters are significantly different and values with no superscript letters are statistically different from those with superscript letters (*p* < 0.05). Values with the same superscript letters do not differ from each other; values without superscript letters do not differ from each other.

**Table 2:** Distribution of antibiotic choice as reported by Lithuanian dental practitioners by age group.

Antibiotic	Age Groups			
	A (≤35 Years) % (n)	B (36–50 Years) % (n)	C (≥51 Years) % (n)	Total % (n)
Amoxicillin	90.3 (187) ^a^	79.9 (179)	73.2 (153)	81.1 (519)
Amoxicillin with clavulanic acid	52.2 (108)	50.4 (113)	53.6 (112)	52 (333)
Clindamycin	2.9 (6)	3.1 (7)	5.3 (11)	3.8 (24)
Doxycycline	0.5 (1)	0.4 (1)	4.8 (10) ^a^	1.9 (12)
Azithromycin	3.9 (8)	2.2 (5)	2.9 (6)	3 (19)
Metronidazole	1.9 (4)	3.6 (8)	3.3 (7)	3 (19)
Cefuroxime	1.0 (2)	1.3 (3)	1.4 (3)	1.3 (8)

^a^—Statistically significantly different from the other two age groups (*p* < 0.05).

**Table 3:** Distribution of the preferences regarding duration of antimicrobial treatment as reported by Lithuanian dentists by their specialties.

	General Dentist % (n)	Oral Surgeon % (n)	Endodontist % (n)	Periodontist % (n)	Prosthodontist % (n)	Other % (n)	Of All Respondents % (n)
3 days	0.8 (4)	0.0 (0)	2.5 (1)	5.0 (1)	0.0 (0)	0.0 (0)	0.9 (6)
5 days	33.2 (165)	22.9 (8) ^a^	42.5 (17) ^b^	20.0 (4) ^a^	37.1 (13) ^b^	45.0 (9) ^b^	33.4 (216)
7 days	61.8 (307) ^a^	68.6 (24) ^b^	45.0 (18)	60.0 (12) ^a^	57.1 (20) ^a^	55.0 (11) ^a^	60.6 (392)
10 days	2.2 (11)	2.9 (1) ^a^	0.0 (0)	15.0 (3) ^b^	0.0 (0)	0.0 (0)	2.3 (15)
Until symptoms disappear	2.0 (10)	5.7 (2)^a^	10.0 (4)^a^	0.0 (0)	5.7 (2)^a^	0.0 (0)	2.8 (18)

Within the same row, values with different superscript letters are significantly different and values with no superscript letters are statistically different from those with superscript letters (*p* < 0.05). Values with the same superscript letters do not differ from each other; values without superscript letters do not differ from each other.

**Table 4:** Distribution of the preferences regarding duration of antimicrobial treatment as reported by Lithuanian dentists by age groups.

Antimicrobial Treatment Duration	Age Group			
	A (≤35 Years) % (n)	B (36–50 Years) % (n)	C (≥51 Years) % (n)	Total % (n)
3 days	1.4 (3)	0.0 (0)	1.4 (3)	0.9 (6)
5 days	21.3 (44) ^a^	32.6 (73)	46.9 (98)	33.6 (215)
7 days	72.9 (151) ^a^	62.5 (140)	46.9 (98)	60.8 (389)
10 days	1.9 (4)	0.9 (2)	3.3 (7)	2 (13)
Until symptoms disappear	2.4 (5)	4.0 (9)	1.4 (3)	2.7 (17)

^a^—Statistically significantly different from the other two age groups (*p* < 0.05).

**Table 5:** Distribution of antibiotic choice for patients allergic to β-lactam antibiotics as reported by Lithuanian dentists categorized by their specialties.

Antibiotic	General Dentist % (n)	Oral Surgeon % (n)	Endodontist % (n)	Periodontist % (n)	Prosthodontist % (n)	Other % (n)	Total % (n)
Clindamycin	61.2 (304)	60.0 (21)	70.0 (28)	65.0 (13)	65.7 (23)	80.0 (16)	62.6 (405)
Azithromycin	16.7 (83)	25.7 (9)	15.0 (6)	15.0 (3)	22.9 (8)	10.0 (2)	17.2 (111)
Metronidazole	8.7 (43)	2.9 (1)	2.5 (1)	5.0 (1)	8.6 (3)	15.0 (3)	8.0 (52)
Erythromycin	14.9 (74)	28.6 (10) ^a^	7.5 (3)	5.0 (1)	22.9 (8) ^a^	10.0 (2)	15.1 (98)
Tetracycline	4.2 (21)	5.7 (2)	0.0 (0)	5.0 (1)	8.6 (3)	0.0 (0)	4.2 (27)

^a^—Statistically significantly different from the other specialists (*p* < 0.05).

**Table 6:** Distribution of antibiotic choice for patients allergic to β-lactam antibiotics as reported by Lithuanian dentists categorized by age groups.

Antibiotic	Age Group			Total % (n)
	A (≤35 Years) % (n)	B (36–50 Years) % (n)	C (≥51 Years) % (n)	
Clindamycin	74.4 (154) ^a^	65.2 (146)	47.8 (100)	62.5 (400)
Azithromycin	13.0 (27) ^a^	17.0 (38)	21.1 (44)	17 (109)
Erythromycin	12.6 (26)	15.2 (34)	17.2 (36)	15 (96)
Metronidazole	8.7 (18)	7.1 (16)	8.1 (17)	8 (51)
Tetracycline	1.9 (4)	2.2 (5)	8.1 (17) ^a^	4.1 (26)

^a^—Statistically significantly different from the other two age groups (*p* < 0.05).

**Table 7:** Antibiotic prophylaxis administered for dental procedures as reported by Lithuanian dental practitioners categorized by specialties.

Pathology	General Dentist % (n)	Oral Surgeon % (n)	Endodontist % (n)	Periodontist % (n)	Prosthodontist % (n)	Other % (n)	Of All Respondents % (n)
Immune suppression due to systemic pathology when recommended by physician	74.8 (372)	85.7 (30)	77.5 (31)	90.0 (18)	77.1 (27)	70.0 (14)	76.0 (492)
Patients at risk of IE after consultation with cardiologist	86.3 (429)	94.3 (33)	95.0 (38)	100.0 (20)	85.7 (30)	80.0 (16)	87.5 (566)
Patients who have undergone arthroplasty within 3 months	46.3 (230)	71.4 (25) ^a^	50.0 (20) ^a^	60.0 (12) ^a^	42.9 (15)	60.0 (12) ^a^	48.5% (314)
Patients in need of head and neck radiotherapy	20.7 (103)	51.4 (18) ^a^	30.0 (12) ^b^	55.0 (11) ^a^	25.7 (9)	35.0 (7) ^a^	24.7 (160)
Patients in need of bisphosphonate therapy	19.9 (99)	57.1 (20) ^a^	37.5 (15) ^a^	55.0 (11) ^a^	20.0 (7) ^b^	20.0 (4) ^b^	24.1 (156)

Within the same row, values with different superscript letters are significantly different and values with no superscript letters are statistically different from those with superscript letters (*p* < 0.05). Values with the same superscript letters do not differ from each other; values without superscript letters do not differ from each other.

**Table 8:** The profile of antibiotic prophylaxis according to respondents’ age groups.

Pathology	Age Groups			Total % (n)
	A (≥35 Years) % (n)	B (36–50 Years) % (n)	C (≤51 Years) % (n)	
Immune suppression due to systemic pathology when recommended by physician	82.6 (171)	77.7 (174)	67.9 (142) ^a^	76.1 (487)
Patients at risk of IE after consultation with cardiologist	90.3 (187)	89.7 (201)	82.3 (172) ^a^	87.5 (560)
Patients who have undergone arthroplasty within 3 months	61.4 (127)	50.4 (113) ^b^	33.0 (69) ^a^	48.3 (309)
Patients in need of head and neck radiotherapy	32.4 (67)	26.8 (60)	13.4 (28) ^a^	24.4 (155)
Patients in need of bisphosphonate therapy	26.6 (55)	26.8 (60)	17.7 (37) ^a^	23.8 (152)

Within the same row, values with different superscript letters are significantly different and values with no superscript letters are statistically different from those with superscript letters (*p* < 0.05). Values with the same superscript letters do not differ from each other; values without superscript letters do not differ from each other.

## Data Availability

The datasets analyzed during the study are available from the corresponding author upon reasonable request.

## Supplementary Materials

The following supporting information can be downloaded at: https://www.mdpi.com/article/10.3390/medicina60111745/s1, Questionnaire S1: Antibiotics prescription for treatment and prevention of odontogenic infections.
