# Supplementary material for: Antibiotic Prescription for Treatment and Prevention of Odontogenic Infections: A Cross-Sectional Survey of Lithuanian Dentists
Source: Medicina (Kaunas). 2024 Oct 24;60(11):1745. doi: 10.3390/medicina60111745 (PMC11596110; doi:10.3390/medicina60111745)
Supplement: Supplementary file 1 [file medicina-60-01745-s001.zip › medicina-3249494-supplementary.pdf]

1. What antibiotic and in what dose do you prescribe to an adult patient without an allergy to antibiotics? Multiply answers possible.

   Penicillin V 1000000TV 3xd
   Amoxicillin 500 mg 3xd
   Amoxicillin 1000 mg 2xd
   Amoxicillin 1000 mg 3xd
   Amoxicillin+Clavulanic acid 500+125 mg 3xd
   Amoxicillin+Clavulanic acid 875+125 mg 2xd
   Clindamycin 300 mg 3xd
   Clindamycin 600 mg 3xd
   Doxycyclin 200 mg 1xd
   Doxycyclin 100 mg 1xd
   Azithromycin 250 mg 1xd
   Azithromycin 500 mg 1xd
   Metronidazole 250 mg 3xd
   Metronidazole 500 mg 3xd
   Cefuroximum 250mg 2xd
   Cefuroximum 500mg 2xd

2. What duration of antibiotic therapy do you usually prescribe for the treatment of odontogenic infection, when indicated? Multiply answers possible.

   3 days
   5 days
   7 days
   10 days
   Until symptoms disappear

3. Which antibiotic do you choose for adult patients with an allergy to penicillin? Multiply answers possible.

   Clindamycin
   Azithromycin
   Metronidazole
   Erythromycin
   Tetracycline

4. When do you prescribe antibiotics?
   Possible answers: always; often; sometimes; seldom; never.

   1.Symptomatic irreversible pulpitis
   2. Pulp necrosis
   3. Symptomatic apical periodontitis
   4. Chronic apical abscess

5. Acute apical abscess without systemic involvement
6. Acute apical abscess in case of immune suppression
7. Acute apical abscess with systemic involvement
8. Rapidly progressing (within 24 h) infection
9. Avulsion
10. Dental trauma
11. Postoperative pain
12. Dental root perforation
13. Constant exudation during treatment
14. 1Pericoronitis
15. After dental extraction
16. After an incision
17. Routinely before implantation
18. After implantation

5. When do you prescribe antibiotic prophylaxis before root canal treatment or surgical procedures? Multiply answers possible.

   Immune suppression due to systemic pathology when recommended by physician
   Patients at risk for IE after consultation with cardiologist
   Patients who have undergone arthroplasty within 3 months
   Patients in need of head and neck radiotherapy
   Patients in need of bisphosphonate therapy
